# Is Khat (Catha edulis) chewing a risk factor for periodontal diseases? A systematic review

**DOI:** 10.4317/jced.54163

**Published:** 2017-10-01

**Authors:** Butchibabu Kalakonda, Sadeq-Ali Al-Maweri, Hashem-Motahir Al-Shamiri, Anum Ijaz, Shukri Gamal, Esam Dhaifullah

**Affiliations:** 1 BDS, MDS, Lecturer, Department of Preventive Dental Sciences, AlFarabi Colleges, Riyadh, Saudi Arabia; 2 BDS, MSc, PhD, Assistant Professor, Department of Oral Medicine and Diagnostic Sciences, AlFarabi Colleges, Riyadh, Saudi Arabia; Department of Oral Medicine, Faculty of Dentistry, Sana’a University, Yemen; 3 BDS, MSc, Lecturer, Department of Oral and Maxillofacial Surgery, AlFarabi Colleges, Riyadh, Saudi Arabia; 4 BDS, General Practitioner, AlFarabi Colleges, Riyadh, Saudi Arabia; 5 BDS, PhD, Assistant Professor, Department of Preventive Dental Sciences, AlFarabi Colleges, Riyadh, Saudi Arabia; 6 BDS, PhD, Assistant Professor, Department of Preventive Dental Sciences Al-Farabi Colleges, Riyadh, Saudi

## Abstract

**Background:**

Khat (Catha edulis) chewing is a highly prevalent habit in the Arabian Peninsula and East Africa, and has recently spread to Western countries. The association between khat chewing and oral mucosal lesions is well documented in the literature. However, there is no concrete evidence on the association between khat chewing and periodontal disease. The purpose of this systematic review was to analyze the influence of khat chewing on periodontal health.

**Material and Methods:**

A literature search of PubMed, Scopus and Web of Sciences databases was carried out to identify relevant articles published from 1990 to May 2017. The inclusion criteria were all clinical studies that assessed the relationship between khat chewing and periodontal disease.

**Results:**

The search yielded 122 articles, of which 10 were included in this systematic review. Most of the studies exhibited a positive correlation between khat chewing and periodontal disease.

**Conclusions:**

Altogether, the analysis of the current evidence reveals that khat chewing is destructive to the periodontium and enhances the risk of periodontal disease progression. However, due to variability of studies, more longitudinal case-controlled studies are highly warranted to establish a causal relation between khat chewing and periodontal disease.

** Key words:**Khat chewing, periodontal health, periodontal disease, risk factor.

## Introduction

Khat or Qat (Catha Edulis) is an evergreen plant that is widely cultivated in Yemen and some parts of East Africa and Southern Arabia (1). The native population of these regions chew its leaves for its stimulating and euphoric effects attributed to cathinone, an amphetamine-like stimulant (1,2). Khat chewing habit is particularly more prevalent in Yemen, Kenya, Ethiopia, Djibouti, Eritrea, Somalia, and South Saudi Arabia (2-4). In recent years khat chewing has spread with immigration to Europe, USA and Australia, despite prohibition by many countries (5,6).

Khat chewing is predominantly a male habit, although the number of women indulging in this habit is on the rise. People usually practice this habit in special social gatherings, known as khat session, which continues for several hours a day. Typically, the habit involves inserting and chewing fresh khat leaves, forming a bolus that is retained in the lower buccal vestibule against the check one side, or rarely, on both sides (2,3). The juice is swallowed and partially expectorated while the quid is ejected at the end of the session.

Khat chewing has potentially several adverse systemic health effects including cardiovascular disorders, liver and gastrointestinal disturbances, renal toxicity, and psychosis (7). Long term khat use has also been associated with several oral and dental disorders such as keratotic white lesions, mucosal pigmentation, plasma cell stomatitis, tooth loss, teeth attrition and discoloration, gingival recession, periodontal diseases, and temporomandibular joint disorders (3,5,8,9).

Several studies have investigated the effects of habitual khat chewing on periodontal tissues. Rosenzweig and Smith (10) were the first to propose a possible effect of khat chewing on periodontal tissues when they noticed that Jewish males of Yemeni origin with history of khat chewing before immigration to Israel had higher rates of periodontal diseases compared to other ethnic groups. More recent studies also reported similar results (9,11-13). Mengel and colleagues (13) investigated the effect of khat on periodontal status among 1001 Yemeni adults and found that the Community Periodontal Index of Treatment Needs (CPITN), the clinical attachment loss and the calculus index were significantly higher in khat chewers compared to non-chewers. Moreover, a 2007 large-scale study among 2500 Yemeni Khat chewers found a positive relationship between the frequency and duration of khat chewing and severity of periodontal diseases (11). On the contrary, some studies (14,15) did not find any significant differences in periodontal health status among chewers and non-chewers, and some even demonstrated less gingival inflammation and less plaque accumulation among chewers than non-chewers (15,16). Additionally, some investigators showed that khat has antimicrobial properties and induces microbial shifts compatible with periodontal health (16,17). In light of the above contradictory results, this review aimed to systematically evaluate the evidence on the association between khat chewing and periodontal disease.

## Material and Methods

-Focused question.

The research question was constructed using the guidelines of Preferred Reporting Items for Systematic Reviews and Meta-Analysis (PRISMA) and according to the Participants, Interventions, Control, and Outcomes (PICO) principle. The focused question of interest was “Is Khat (Catha edulis) chewing a risk factor for periodontal disease?”.

-Eligibility Criteria.

Our search included longitudinal studies, case-control studies and cross-sectional studies that assessed the relationship between khat chewing and periodontal diseases with respect to bleeding on probing, probing depth, attachment loss, gingival recession, gingival inflammation and tooth loss. Exclusion criteria were animal studies, experimental studies, *in-vitro* studies, case reports, case series, review papers, letters to the editor, monographs, conference papers, unpublished data and studies published in a language other than English.

-Literature search protocol.

A literature search of PubMed, Scopus, and Web of Knowledge (IS) databases was conducted to identify relevant articles published in English from 1990 up to May 2017, using different combinations of following keywords: Khat, Qat, Catha edulis, periodontitis, gingivitis, periodontal health, periodontal disease, attachment loss and tooth loss.

Titles and abstracts of retrieved studies were screened for eligibility by two independent authors (SA and BK), and all irrelevant studies were excluded. The full texts of apparent relevance was obtained and then read and assessed for inclusion. Moreover, the reference lists of relevant articles were manually searched for additional studies.

-Data extraction:

The following data were extracted from the included articles by two independent authors using a standardized data collection form: authors and year of study, study design, number of subjects, mean age, chewing duration and frequency, variables assessed (like plaque index, gingival index, calculus index, probing depth, attachment level, gingival recession, bleeding on probing) and the main outcomes.

-Assessment of quality:

Critical appraisal of the included studies was performed by two independent authors. Criteria for assessing the quality of studies were adapted from the Strengthening the Reporting of Observational studies in Epidemiology Statement (STROBE) (18). Seven criteria were considered the most important ones in context of this review and were included in the checklist.

The STROBE checklist comprised the following: whether the study design is clearly stated, study participants are fully described, sample size is justified, variables are clearly defined, potential confounders are addressed, outcomes are accurately measured and appropriate statistical analysis tests were used. Each criterion was given a response of either “Yes” or “NO”. Each study could have a maximum score of 7. After the scores were summed, the methodological quality was graded as low (0-3), acceptable (4-5), and high (6-7).

## Results

-Study selection

**Figure 1:**
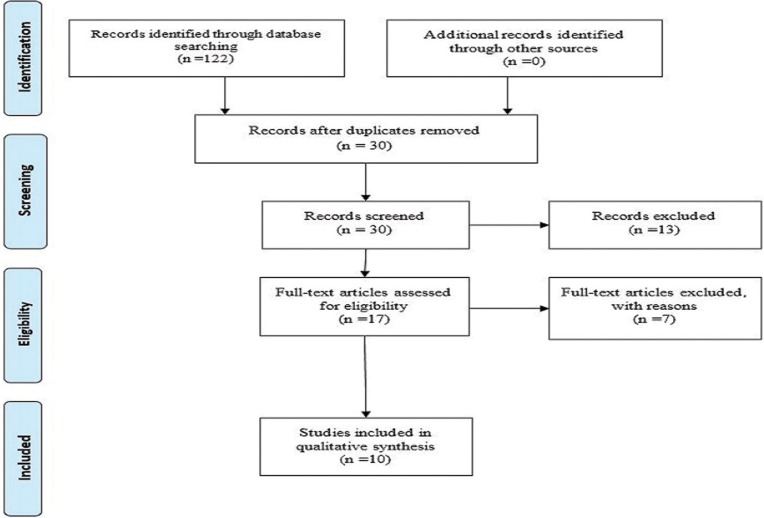
Flow chart of methodology according to PRISMA guidelines.

The search strategy for identification of relevant studies following the PRISMA guidelines is presented in Figure 1. An initial search identified a total of 122 studies obtained from various databases, of which 92 were duplicates. After screening of titles and abstracts, 13 studies were found irrelevant and thus excluded. Only 17 studies fulfilled the eligibility for full-text evaluation. Of these, 7 studies have been excluded for various reasons, and the remaining 10 (2,9,11-15,19-21) studies were included in the systematic review and processed for data extraction.

-General characteristics of the included studies

The characteristics of the included studies are summarized in  Table 1,  Table 1 continue. With respect to the study design, seven studies were cross-sectional (2,11,13,14,19-21) and three were case-control (9,12,15).

**Table 1:**
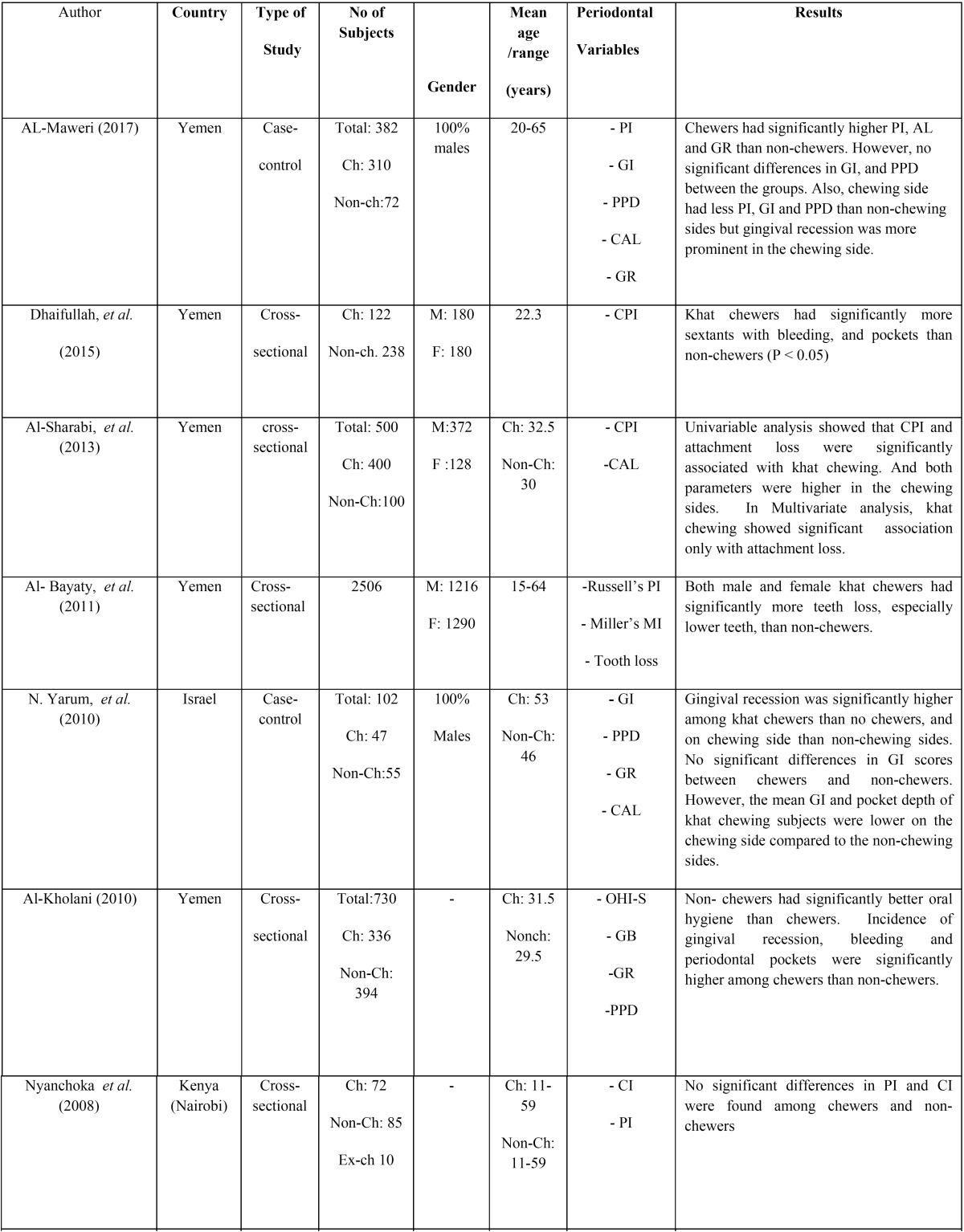
General characteristics of the included studies.

**Table 1 continue:**
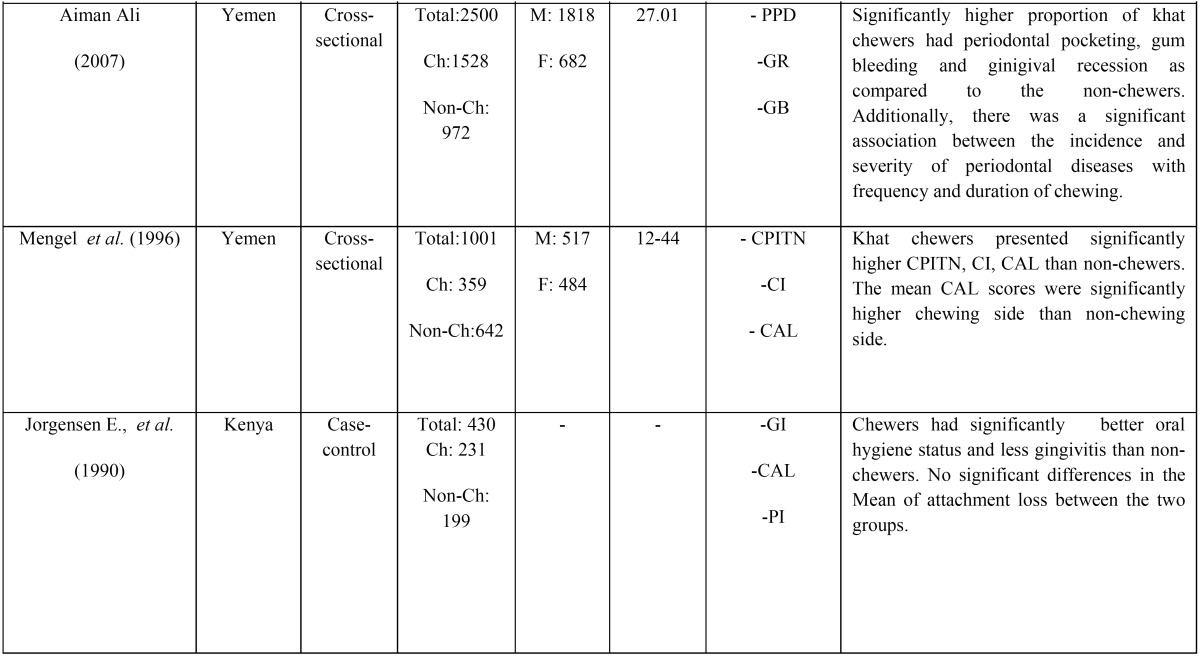
General characteristics of the included studies.

Of the ten studies, seven were conducted in Yemen (2,11-13,19-21,), two in Kenya (14,15) and one in Israel (9). The total number of subjects involved in these studies ranged between 102 and 2506, with an exclusively male predominance. Five studies reported the mean age of the participants, which was between 22.3 and 54 years, with a range of 11-65 years.

-Clinical periodontal parameters investigated

Probing pocket depth (PPD) was reported in seven studies (2,9,11-13,19,21) and clinical attachment loss was reported in five studies (2,9,12,13,15). Three studies (12,14,15) assessed plaque index and gingival index was reported in three studies (9,12,15). Gingival recession was reported in two studies (9,21). One study (20) assessed rate of tooth loss among chewers and non-chewers. All studies assessed periodontal health status clinically.

-Main outcomes of the studies

One study (15) reported that khat chewers had significantly better oral hygiene status and less gingivitis compared to non-chewers whereas one study (14) found no significant differences in PI and CI among chewers and non-chewers. In contrast, majority of the included studies (2,12,13,19,21) have reported that khat chewers had reportedly overall poorer oral hygiene status reflected by their plaque scores and calculus deposits compared to the non-chewers.

Majority of the studies (2,9,11-13,19) reported a significant association between khat chewing and various periodontal parameters such as periodontal pocketing, bleeding upon probing, clinical attachment loss and gingival recession.

Some studies (2,9,12) reported less gingival inflammation and less plaque accumulation but higher attachment loss in the chewing sides compared to the non-chewing sides of khat chewers.

AL-Maweri and Al-Akhali (12) in their case-control study found that khat chewers had a higher overall mean plaque scores along with clinical attachment loss and gingival recession compared to non-chewers. However, it was observed that there were no significant differences in gingival index and probing pocket depth scores among the groups. It was also evident that the duration and the frequency of chewing had a significant effect on further attachment loss and gingival recession. The authors observed that chewing side of khat chewers had less PI, GI and PPD than non-chewing sides but gingival recession was more prominent in the chewing side.

In a cross-sectional study, Al-sharabi along with his colleagues (2) investigated the effect of khat chewing as an independent risk factor for periodontitis. Four hundred khat chewers and 100 non-chewers aged 20 to 50 years were included in his study. Periodontal assessment was done using community periodontal index and clinical attachment loss. After adjusting confounding variables such as smoking, level of education and oral hygiene, it was found that khat chewers exhibited more clinical attachment loss compared to non-chewers.

In a large-scale study (n=2500), Ali (11) evaluated the effect of khat chewing habit as a causative factor for periodontal disease in Yemen society. Detailed questionnaire and periodontal assessment was carried out. The study results revealed that khat chewers presented with increased periodontal pockets and gingival recession compared to non-chewers, and the severity of periodontal destruction was significantly associated with duration and frequency of the habit.

Al-Bayaty (20) assessed the effect of khat chewing on tooth loss among 2506 Yemeni adults, of whom 1206 were males and 1290 were females. They found that male and female khat chewers had significantly more tooth loss than non-chewers. The authors concluded that khat chewing is one of the risk factor for tooth loss.

Quality of the included studies:

The results of the STROBE-based quality analysis are presented in  Table 2. The total quality score ranged from 3-7. The most common shortcomings among all studies were the lack of sample size calculation and failure to address confounding factors particularly smoking. The overall quality of included studies assessing the association between khat chewing and periodontal diseases was good; nevertheless, small sample sizes and the failure to address potential confounding factors limit the application of these study outcomes.

**Table 2:**
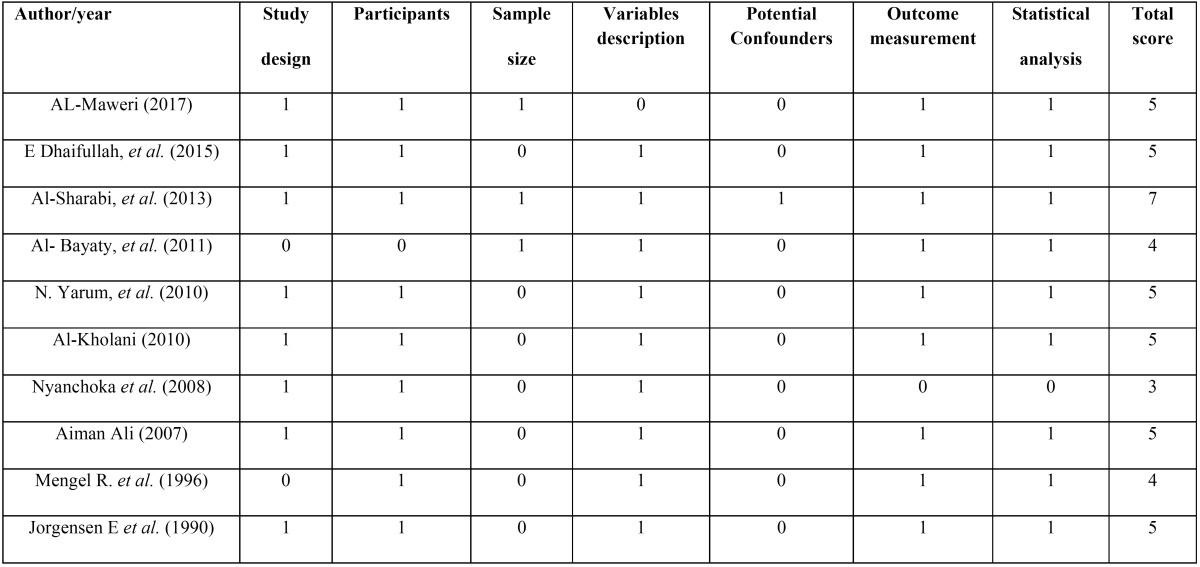
STROBE -based quality analysis of the included studies.

## Discussion

The present systematic review was envisioned to explore the association of khat chewing and periodontal disease. Analysis of the available evidence showcases that khat chewing has substantial detrimental and destructive effects on the periodontal health, and further portray it as a risk factor for periodontal disease.

The term “periodontal diseases” encompasses both gingivitis and various forms of periodontitis. The progression of periodontal disease is influenced by various risk factors. Among these risk factors there has been a strong evidence associating smoking and smokeless tobacco products as significant risk factors in the progression of periodontal disease that has been established in various hallmark studies (22,23). Following these events, recently researchers have evinced interest to link khat chewing with periodontal disease.

The influence of khat chewing on periodontal disease was assessed through its effect on various periodontal parameters; such as gingival inflammation, gingival recession, attachment loss and probing pocket depth. Summation of the evidence available reveals that khat chewing has a substantial effect on all these parameters.

Determination of the observed effects of khat chewing on gingival inflammation was taken up first with the evaluation of its so-called ‘anti-plaque and anti-gingivitis properties’ through gingival and plaque indices. Interestingly, interpretations from some of the studies revealed that khat chewers had less gingival inflammation and plaque accumulation reflected through lower gingival index scores, plaque scores and bleeding on probing (9,15). This has been partly attributed to the chemical composition (tannins) in khat which has an astringent effect and control plaque formation (14). The anti-plaque and anti-gingivitis properties of khat chewing have also been perceived by its ability to influence the quantity and quality of dental plaque. Khat chewing by its mechanical cleansing action on the chewing side aids in the reduction of the quantity of dental plaque and it influences the quality of dental plaque by promoting a microbial shift from gingivitis to an environment compatible with periodontal health (1,12,24,25).

Furthermore, analysis of the current evidence indicates that khat chewing had a detrimental effect on the periodontal parameters; pocket depth, gingival recession and clinical attachment loss. Though pocket depth has been a cardinal symptom for periodontal disease, it does not account for possible recession of the gingival margin. Further, increased prevalence of gingival recession among khat chewers, probably suggests that reliance on pocket depth predominantly would have underestimated disease severity in khat chewers. Hence, gingival recession along with attachment loss were considered as the primary determinants representative of periodontal disease in our review. Aggregating the results from various studies, it was evident that khat chewers had significantly more attachment loss and gingival recession (2,9,12,13,21). This could most probably be attributed to the intensity and the duration of the habit resulting in chronic trauma associated with high occlusal load leading to vertical impaction into the periodontium resulting in gingival recession and clinical attachment loss.

A key concern in the present review was pooling of evidence and presentation of the effects of khat chewing on periodontal disease without taking into consideration other risk factors such as smoking, dietary habits and oral hygiene practices that act as potential confounders and have a deceptive influence of khat chewing on periodontal disease outcomes. Among the ten studies included in this review, only one study (2) reported control of potential confounders. Longitudinal observational studies with exclusion of confounding variables is suggested to establish a true causal relationship between khat chewing and periodontal disease.

The present systematic review had some limitations. The primary limitation was related to the fact that most of the included studies were confined to the Yemeni population, where habitual khat chewing is deep-rooted into their culture (2,12,13,19-21). Hence, generalizing the results based on outcomes obtained in one country is a shortcoming in this review. Second, most of the studies had predominantly male khat chewers. The lower use of khat among females could be attributed to taboos and culture restrictions. Furthermore; methodological variations in the study designs, varied sample sizes, inadequate measurement of periodontal disease and predominant reliance on cross-sectional studies were limitations of this review that restricted us from performing a meta-analysis.

## Conclusions

In conclusion, analysis of the available evidence reveals that khat chewing has a significant influence with higher risk of periodontal disease progression reflected by increased attachment loss and gingival recession. These detrimental effects of khat chewing on the periodontium outweigh its beneficial antimicrobial properties.

Future research to strengthen the evidence related to the effect of khat chewing on periodontal disease should include studies from different countries with a prospective cohort and case-control design that control all potential confounding factors.

## References

[1] Al-Hebshi NN, Skaug N (2005). Khat (Catha edulis)-an updated review. Addict Biol.

[2] Al-Sharabi AK, Shuga-Aldin H, Ghandour I, Al-Hebshi NN (2013). Qat chewing as an independent risk factor for periodontitis: a cross-sectional study. Int J Dent.

[3] Al-Maweri SA, Alaizari NA, Al-Sufyani GA (2014). Oral mucosal lesions and their association with tobacco use and qat chewing among Yemeni dental patients. J Clin Exp Dent.

[4] Awadalla NJ, A Suwaydi H (2017). Prevalence, determinants and impacts of khat chewing among professional drivers in Southwestern Saudi Arabia. East Mediterr Health J.

[5] Hijazi M, Jentsch H, Al-Sanabani J, Tawfik M, Remmerbach TW (2016). Clinical and cytological study of the oral mucosa of smoking and non-smoking qat chewers in Yemen. Clin Oral Investig.

[6] Kassim S, Jawad M, Croucher R, Akl EA (2015). The epidemiology of tobacco use among khat users: a systematic review. Biomed Res Int.

[7] Al-Motarreb A, Al-Habori M, Broadley KJ (2010). Khat chewing, cardiovascular diseases and other internal medical problems: the current situation and directions for future research. J Ethnopharmacol.

[8] Al-Maweri SA, Al-Jamaei AA, Al-Sufyani GA, Tarakji B, Shugaa-Addin B (2015). Oral mucosal lesions in elderly dental patients in Sana'a, Yemen. J Int Soc Prev Community Dent.

[9] Yarom N, Epstein J, Levi H, Porat D, Kaufman E, Gorsky M (2010). Oral manifestations of habitual khat chewing: a case-control study. Oral Surg Oral Med Oral Pathol Oral Radiol Endod.

[10] Rosenzweig KA, Smith P (1996). Periodontal health in various ethnic groups in Israel. J Periodontal Res.

[11] Ali AA (2007). Qat habit in Yemen society: a causative factor for oral periodontal diseases. Int J Environ Res Public Health.

[12] Al-Maweri SA, AlAkhali M (2017). Oral hygiene and periodontal health status among khat chewers. A case-control study. J Clin Exp Dent.

[13] Mengel R, Eigenbrodt M, Schünemann T, Florès-de-Jacoby L (1996). Periodontal status of a subject sample of Yemen. J Clin Periodontol.

[14] Nyanchoka IN, Dimba EA, Chindia ML, Wanzala P, Macigo FG (2008). The oral and dental effects of khat chewing in the Eastleigh area of Nairobi. J Kenya Dent Assoc.

[15] Jorgensen E, Kaimenyi JT (1990). The status of periodontal health and oral hygiene of Miraa (catha edulis) chewers. East Afr Med J.

[16] Al-hebshi NN, Al-Akhali MS (2010). Experimental gingivitis in male khat (Catha edulis) chewers. J Int Acad Periodontol.

[17] Al-Alimi A, Taiyeb-Ali T, Jaafar N, Noor Al-hebshi NN (2015). Qat Chewing and Periodontal Pathogens in Health and Disease: Further Evidence for a Prebiotic-Like Effect. Biomed Res Int.

[18] Vandenbroucke JP, von Elm E, Altman DG, Gøtzsche PC, Mulrow CD, Pocock SJ (2007). Strengthening the Reporting of Observational Studies in Epidemiology (STROBE): explanation and elaboration. Epidemiology.

[19] Dhaifullah E, Al-Maweri SA, Al-Motareb F, Halboub E, Elkhatat E, Baroudi K (2015). Periodontal Health Condition and Associated Factors among University Students, Yemen. J Clin Diagn Res.

[20] Al-Bayaty FH, Ali NW, Bulgiba AM, Masood M, Hussain SF, Abdulla MA (2011). Tooth mortality in khat and non khat chewer in Sana'a Yemen. Sci Res Essay.

[21] Al-Kholani AI (2010). Influence of khat chewing on periodontal tissues and oral hygiene status among Yemenis. Dent Res J (Isfahan).

[22] Bergstrom J (1989). Cigarette smoking as risk indicator in chronic periodontal disease. Community Dent Oral Epidemiol.

[23] Robertson PB, Walsh M, Greene J, Ernster V, Grady D, Hauck W (1990). Periodontal effects associated with the use of smokeless tobacco. J Periodontol.

[24] Al-Hebshi NN, Skaug N (2005). Effect of khat chewing on 14 selected periodontal bacteria in sub- and supragingival plaque of a young male population. Oral Microbiol Immunol.

[25] Al-Hebshi NN, Al-Sharabi AK, Shuga-Aldin HM, Al-Haroni M, Ghandour I (2010). Effect of khat chewing on periodontal pathogens in subgingival biofilm from chronic periodontitis patients. J Ethnopharmacol.

